# Long-term consequences of mothers’ and fathers’ wartime deployments: Protocol for a two-wave panel study

**DOI:** 10.1371/journal.pone.0295007

**Published:** 2024-03-18

**Authors:** Shelley M. MacDermid Wadsworth, Dave Topp, Patricia Lester, Valerie Stander, Sharon L. Christ, Shawn Whiteman, Leanne Knobloch

**Affiliations:** 1 Department of Human Development and Family Science, Purdue University, West Lafayette, Indiana, United States of America; 2 Department of Psychiatry and Biobehavioral Sciences, University of California, Los Angeles, California, United States of America; 3 Naval Health Research Center, San Diego, California, United States of America; 4 Department of Human Development and Family Studies, Utah State University, Logan, Utah, United States of America; 5 Department of Communication, University of Illinois, Urbana, Illinois, United States of America; The Quinism Foundation, UNITED STATES

## Abstract

Multiple adjustment difficulties have been associated with children’s exposure to recent parental wartime military deployments, but long-term consequences have not yet been systematically studied. This investigation will assess direct and indirect relationships between exposures to parental deployments early in life and later youth adjustment. Parents’ psychological health and family processes will be examined as mediators, and parents’ and children’s vulnerability and support will be examined as moderators. Archival data will be combined with new data gathered from two children and up to two parents in families where children will be aged 11 to 16 at the first data collection and will have experienced at least one parental deployment, for at least one child prior to age 6. Data are being gathered via telephone interviews and web-based surveys conducted twice one year apart. Outcomes are indicators of children’s social-emotional development, behavior, and academic performance. Notable features of this study include oversampling of female service members, inclusion of siblings, and inclusion of families of both veterans and currently serving members. This study has potentially important implications for schools, community organizations and health care providers serving current and future cohorts of military and veteran families.

## Introduction

Multiple short-term adjustment difficulties, including elevated behavior problems [1, 2], risk-taking and suicidal thoughts [3],impairments in academic performance, and difficulties with peers [4] have been associated with recent exposure to parental wartime deployment among the approximately two million children of U.S. military personnel who have served in ongoing conflicts in Iraq, Afghanistan, or other combat zones since 2001 [5] Exposures to parental wartime deployments may have long-term consequences for children, particularly when exposures occur early in life [6, 7], but these have not yet been systematically studied. These early adverse experiences could challenge the foundation for children’s later emotion regulation and relationship functioning [8, 9]. The consequences of early exposures to adversity may be particularly evident during adolescence [10, 11] when young people make decisions about substance use, risky behavior, and peer relationships that are highly consequential for their later functioning [9]. Evidence suggests that substantial percentages of these youth are troubled–in 2013, for example, military children in California in 9th or 11th grades were 43% more likely than civilian peers to have considered suicide [12]. There is considerable variability, however, in children’s responses to parental deployments, highlighting a critical knowledge gap in understanding explanatory factors associated with both negative and positive adaptation [10, 13].

### Model for the proposed study

**Fig 1:**
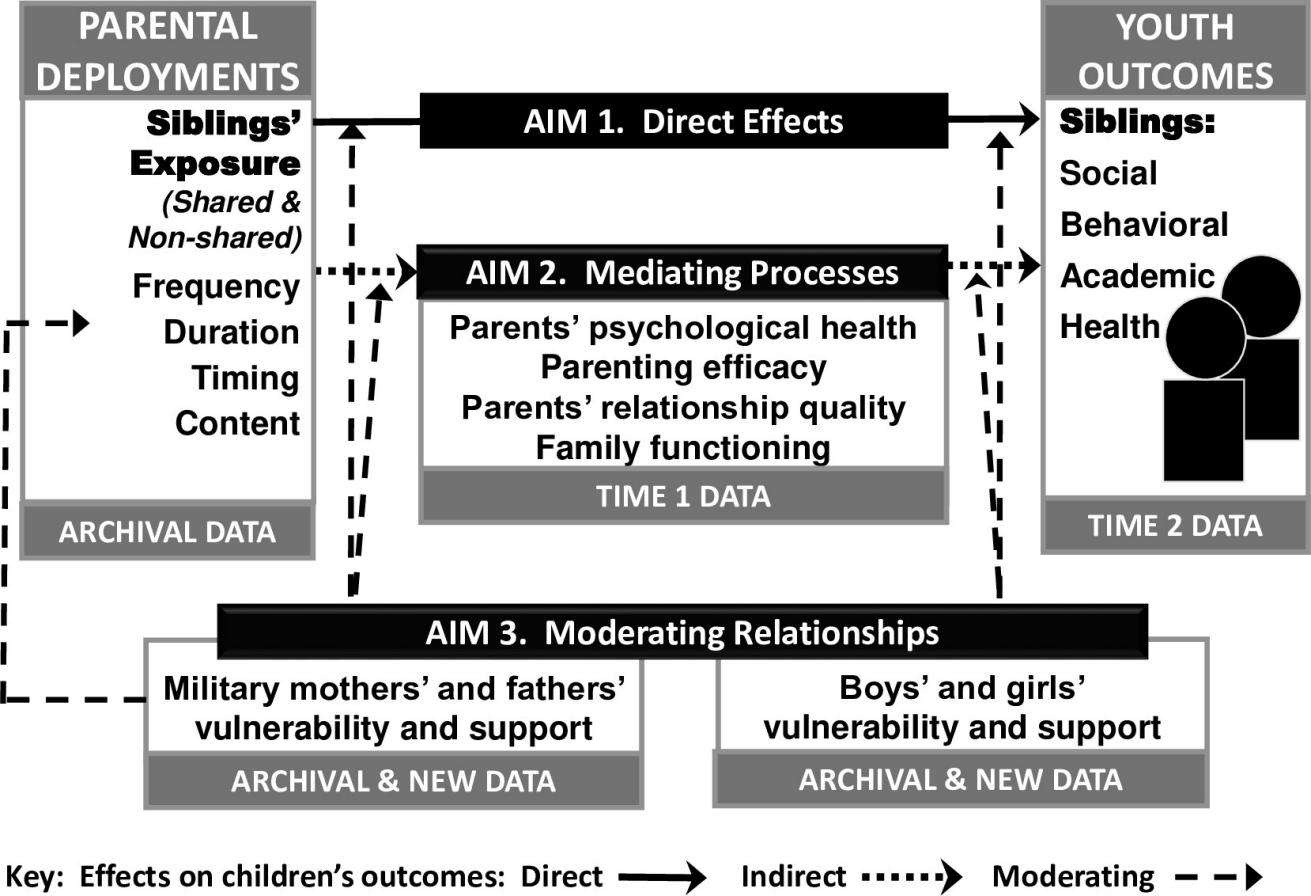
Conceptual model.

Theory and limited existing evidence suggest that exposure to parental deployments early in life is directly related to children’s later outcomes (see Aim 1 in Fig 1) [6, 14].Separation from a parent is well-established as a consequential adverse experience for children, with implications for later psychological [15, 16] and behavior problems [15]. In one study, sleep disturbances in adulthood were linked to separations lasting only one month during the first year of life [17]. Wartime separation from a military parent may be especially distressing, as children experience worry and fear about the deployed parent as well as concern about their at-home parent [18]. One of the preliminary studies for this research was the first to examine the implications of exposure timing in the lives of young children, finding for example that prenatal exposure to parental deployment was significantly related to peer problems among 6 to 10 year olds [19]. These direct relationships may differ, however, across siblings in the same family due to personal characteristics or differences in the timing of exposure. This study will be the first to address this issue.

The mediated relationships between exposure to parental deployment and youth outcomes in the model for this study are guided by research about risk and resilience in families. Four possible mechanisms will be tested in this study (see Aim 2 in Fig 1). For both returning service members and at-home partners, parents’ psychological health (i.e., during- or post-deployment depression, anxiety, PTSD, or substance use) is associated with multiple adjustment difficulties in children [20–22] and similar relationships are expected here. Other indirect pathways travel through family relationships, which have been studied less in relation to deployment than other mechanisms. Here, guided by findings that ‘risky’ families [23, 24] and resilience in families [25, 26] have substantial effects on children’s long-term outcomes, youth outcomes are expected to be worse when deployment is associated with parenting behavior that is hostile or emotionally cold rather than warm and responsive [23, 24], and better when the reverse is true. Existing research indicates that parents in military families report more difficulties, and in some cases compromised caregiving quality, during and following deployment [5, 27, 28].

With regard to marital or partner relationships, the Institute of Medicine [5] concluded the evidence is ‘strong’ that service members returning from combat deployments are at elevated risk of marital conflict and violence [92 93]. Research in the general population indicates that interparental conflict can disrupt children’s long-term outcomes, resulting in elevated emotional insecurity and internalizing and externalizing problems [29]. Although research with military families on this topic is rare, one recent study found that, during reintegration, interparental conflict was negatively related to youth’s self-efficacy and well-being and positively associated with their anxiety and depressive symptoms [30]. More positive youth outcomes are thus expected when there is less conflict, more constructive communication, and better cooperation between parents.

With regard to family functioning, family resilience theory suggests that resilience is more likely to occur in the aftermath of adversity when multiple family members perceive that their family is cohesive, well-organized, able to communicate and solve problems effectively, and shares a sense of optimism and confidence that life has meaning [26, 31]. Importantly, this study will advance research on military families by assessing sibling relationships as a critical family relationship context. Past research with civilian families has found that positive family interaction mediates relationships between risk factors (e.g. parental depressed mood, marital conflict, cumulative risk) and children’s adjustment [32, 33]. Studies of civilian populations highlight how siblings’ relationships with one another constitute unique risk and resilience factors for youth adjustment [34]. In this study, more positive youth adjustment is expected when multiple family members—with specific attention to siblings–report and demonstrate that the family is functioning well [29].

To date, most evidence about moderating relationships involving deployments and children’s outcomes (see Aim 3 in Fig 1) has focused on characteristics of individual children (e.g., age) or service members (e.g., active vs. reserve), with mixed results [6]. Deployments do not occur in isolation, however. The Army STARRS [35], Millennium Cohort [36], and other studies [37] suggest that prior exposure to adversity moderates service members’ later vulnerability to traumatic deployment experiences. As a result, the sample for this study was intentionally selected based on military parents’ exposures to adverse experiences prior to deployment. Similarly, children’s exposure to parental deployment may be an isolated experience, or it may join—or launch—a ‘caravan’ of other adverse experiences shaping later adjustment, helping to explain diversity in children’s outcomes [38]. Parents’ and children’s lifetime exposures to adversity and perceptions of support will be treated as potential moderators of relationships between deployment and intervening or outcome variables. Comparing two siblings in each family will allow separation of child-specific factors from those of deployment or parental factors in relation to children’s outcomes, a significant contribution to the risk and resilience literature.

This study also is designed to permit consideration of both parent and youth sex as moderators of the relationships between early exposure to parental deployments and later youth adjustment. There are good reasons to expect that mothers’ deployments may be especially impactful or that boys and girls may be differentially vulnerable, but existing evidence is mixed.

Consistent with Masten and Narayan’s [39] recognition that the developmental timing of adverse experiences is consequential, deployments, life events, and other experiences will be coded in ‘child time’ (i.e., months since birth), including exposures occurring prenatally, as well as timing relative to deployment. More positive outcomes are expected when exposures began later and were less frequent or prolonged, and/or when parents experienced fewer traumatic experiences during deployment. The controlled heterogeneity of deployment timing in this study will help to provide greater clarity about the role of child age in these effects, thus addressing the lack of consistency in previous findings in relation to deployment effects.

### Aim 1: To assess direct relationships between the timing, frequency, duration and nature of siblings’ early exposures to parental deployments and later youth adjustment

Youth whose deployment exposures began earlier in childhood, were more frequent or prolonged, and/or involved more traumatic [40] parental experiences are hypothesized to display less positive adjustment, and youth exposed to deployments with fewer such characteristics will display more positive adjustment. We will examine how these direct relationships differ between siblings, providing more sensitive tests than previously possible of the degree to which youth outcomes are a function of deployment, family, and child factors.

### Aim 2: To assess the role of parents’ psychological health and family processes in mediating relationships between siblings’ early exposures to parental deployment and later youth adjustment

Mediation is hypothesized to occur through four pathways: a) parents’ psychological health; b) parenting efficacy; c) the quality of parents’ relationship with one another; and d) the quality of family functioning, with particular attention to the quality of sibling relationships, each of which is expected to be positively related to youth adjustment.

### Aim 3: To assess the moderating role of parent and sibling sex, and vulnerability and support in the relationship between siblings’ early exposures to parental deployment and later youth adjustment

Relationships between parental deployments and youth adjustment are expected to be stronger in the presence of greater vulnerability (i.e., adverse experiences, mental health problems) and less support (i.e., availability and use of formal and informal support) prior to and following deployment. Parent and youth sex will be examined as potential moderators of the impact of the relationship between exposure to parental wartime deployment and outcomes.

Existing studies of military children are limited by significant design constraints, such as focusing only on current or recent deployments, which leaves unanswered questions about long-term consequences [10]. Few studies have included data about military parents’ experiences before deployment, which may lead researchers to attribute to deployments parental factors that not only existed before, but may have conditioned parents’ reactions to deployment. Most studies have relied almost completely on parents’ reports for data about children, which can be influenced by parents’ own symptoms [41]. Few studies have compared the consequences of mothers’ and fathers’ deployments, none in the long term. The proposed study is unique because its combination of archival and new data surmounts these design constraints of existing studies.

## Methods

### Overview of study design

This study will combine archival data with new data gathered on two occasions 12 months apart. Data are being gathered via telephone interviews and supervised self-administered surveys with two siblings and up to two parents in 712 families.

This study is being conducted with oversight by the Institutional Review Board at the Naval Health Research Center (Protocol Number: NHRC.2019.0019). The Institutional Review Board at Purdue University approved deferral to the Naval Health Research Center IRB (Purdue IRB-2020-440) and the Army Human Research Protections Office concurred with the determination made by the Naval Health Research Center IRB (HRPO; Log Number: E02093). The study also was reviewed by the Department of Defense Office of People Analytics and issued DoD Survey License Exemption (#9)—Exempt#-0100. The Office of Military Family Readiness Policy in the U.S. Department of Defense is serving as the DoD sponsor of data requests. Parents provide written consent for their own and their children’s participation; adolescents provide written assent for their own participation.

Archival data will include demographic, deployment, and medical records from DOD sources. Demographic variables will come from the Defense Enrollment Eligibility Reporting System (DEERS), and will include military career information such as service branch and component, dates of accession and promotion; personal variables such as birthdate, gender, race/ethnicity; and family information such as spouses’ and children’s names, genders, and birthdates; and family contact information.

Deployment history information will come from the Contingency Tracking (CTS) and Personnel Tempo (PERSTEMPO) files, and will include reasons for deployment (e.g., training, contingency operations), dates, locations, and hazardous duty pay status. Medical record data will include health care utilization and diagnoses from the Medical Data Repository (MDR) including mental health diagnoses of both parents since the first deployment of the service member(s); parents’ physical injuries resulting from operations of war and screening and diagnosis of Traumatic Brain Injury; and children’s lifetime outpatient and inpatient visits with documentation of childhood disorders (e.g., Autism) and psychiatric disorders (e.g., mood and anxiety disorders). Outcomes of interest will include multiple indicators of children’s social-emotional development, behavior, academic performance, and health. Analyses will focus on multilevel examinations of direct, mediated, and moderating relationships between early exposure to parental wartime deployment and later youth outcomes. The study has been approved by the Institutional Review Board at the Department of Defense Naval Health Research Center. Data collection began in the spring of 2023, and recruitment is expected to continue for 16 months.

### Sample

Data are being gathered from two siblings and up to two parents in 712 families where children will be aged 11 to 16 at the launch of data collection and living in the continental United States, at least half time with their military parent since the deployment. Children each will have experienced the same parental wartime deployment lasting at least 90 days, prior to age 6 for at least one child. A spouse or partner presently living with the military parent will also be invited to participate; an average of 1.66 parents per family is anticipated. Over 1 million service member parents have been deployed to Iraq and Afghanistan since 2001; 33.5% of their children are in the targeted age range [5, 42], yielding an estimated maximum pool of more than 200,000 families after accounting for families with only one child.

Families are not being excluded by divorce so long as the participating children have lived at least half time with the military parent. Families will be excluded if there is an active ongoing investigation for family violence; if any of the prospective participants has a significant cognitive impairment that would prevent comprehending or responding to questions; if the military parent is deployed during the study period; if only one adolescent is willing to participate; or if the military parent does not fit within a stratum with unfilled spaces.

The sampling frame was constructed using probability methods from DoD personnel records by the Defense Manpower Data Center (DMDC) using information about family members in the Defense Enrollment Eligibility Reporting System (DEERS) and deployments in the Contingency Tracking System (CTS) and PERSTEMPO file. The sample was drawn to ensure representation of service branches, components (active or reserve), and paygrades (officer/enlisted) using a systematic sampling method. To compensate for their low representation in the military [43], female military parents were oversampled to comprise 65% of the sample using explicit stratification [42]. The sample also was stratified according to military parents’ exposure to adverse experiences, compensating for higher nonresponse typical of high risk groups. Based on national data, approximately half the military parents are expected to have experienced at least one of 12 adverse experiences prior to deployment [9]. Based on screening, each family is assigned to gender and risk groups as they enter the study. Interviewers are blind to participant risk group.

### Sample size determination with statistical power calculation

Data will be obtained from 712 families at baseline, with 2 children and up to 2 parents per family (given divorce rates, 1.66 parents per family are estimated). Stratification will be used to over-select female service members and high-risk families such that 456 families will have female military parents and 356 families will be high risk. Power is calculated for three types of analytic samples:

1. A baseline/cross-sectional analysis sample of 712 families and 1,424 children (2 per family), and 1,182 parents (1.66 per family);
2. A panel analysis sample composed of families who participate at both waves of the study. Based on experience and other military studies [44], 75% retention is projected, resulting in 534 families, 1,068 children, and 887 parents at Wave 2;
3. A repeated measures sample, comprising all observations from both waves. With a 75% family retention rate, the sample will include 1,246 (= 712 + 534) repeated family observations and 2,670 repeated child observations, and 2,216 repeated parent observations. The repeated measures sample will be the priority sample for testing study hypotheses.

Effective sample sizes are calculated for a two-level, mixed-effects model per Snijders [45] using an estimated within-family ICC = 0.30. Power is estimated for a standardized regression coefficient in a model with 5 covariates and alpha = 0.05. Effect sizes detectable at .80 power are listed in Table 1.

**Table 1:** Detectable effect sizes.

Direct, Moderation, and Mediation Effects (n = cross-sectional / panel / repeated)	Detectible Standardized Beta		
	Cross- sectional	Panel	Repeated Measures
**1. Family level predictors** (n = 712 / 534 / 1246; effective n = 1095 / 822 / 1917)	0.085	0.098	0.064
**2. Child level predictors** (n = 1424 / 1068 / 2492; effective n = 2034 / 1526 / 3560)	0.063	0.072	0.047
**Simple Effects**			
**3. Family level predictors within risk and sex groups** (n = 356 / 267 / 623; effective n = 548 / 411 / 958)	0.120	0.139	0.091
**4. Child level predictors within risk and groups** (n = 712 / 534 / 1246; effective n = 1017 / 763 / 1780)	0.088	0.102	0.067

Power for tests 1 and 2 in the table apply to direct effects (**Aim 1** paths in Fig 1); mediation effects (products of path coefficients; **Aim 2** paths in Fig 1), and moderating effects (differences in slopes/interactions; **Aim 3** paths in Fig 1); Tests 3 and 4 represent simple slopes for associations within levels of the moderators family risk status and service member sex, which represent the lowest powered tests.

Some cite 0.10 as a “small” effect size for a partial correlation, which is comparable to a standardized beta coefficient [46]. Recent findings about effects of recent deployment (duration, trauma) effects on military youth outcomes gave standardized beta effect estimates in the range of 0.18 to 0.32, on average [44]. These reported effects were controlling for baseline levels in child outcomes and therefore represent effects on residual change, giving confidence that the study is adequately powered. Importantly, power will be further increased through the use of latent variables, which increase measure reliability [47], correlated mediators and outcomes in multivariate analyses [48], and including control variables that explain variance in mediators/outcomes (particularly true in panel analyses). However, power may decrease due to the unequal weighting effect [49].

### Recruitment and consent procedures

After the sampling frame was received in the fall of 2020, public records searches were undertaken to verify and update addresses, and recruitment began, using evidence-informed procedures similar to those previously approved by regulatory authorities for use in studies of military populations (e.g., our preliminary studies, Millennium Cohort Study) [50].

Beginning in the spring of 2023, eligible participants received a series of communications: **A)** A printed postcard indicating that an invitation to participate will be coming; **B)** A hard-copy packet of recruitment materials including an invitation letter and a Frequently Asked Questions brochure inviting the military parent, his or her spouse, and two children to participate. If multiple children are eligible, the youngest two are invited to participate (to minimize the likelihood of ‘aging out’ or leaving home before the end of the study). Separate packets are included for spouses and children, along with a small preincentive [51]. Families are asked to indicate interest by calling an telephone number, sending email to a specified address, returning a reply card, or visiting a website; **C)** A series of email or postcard reminders that include links to the study website; and **D)** A limited sequence of telephone call attempts to invite participation and respond to questions.

#### Parent screening

When a family indicates interest, a screening call is scheduled with the military parent to verify stratification variables and living situation of youth. Screening for adversity consists of asking military parents if they experienced any of 8 specified adverse experiences prior to joining the military. Because participants have not yet given informed consent, they are not asked which specific events occurred or when (detailed questions are asked during the survey and interview). If all criteria are met, and the study has capacity in the appropriate risk stratification group, researchers confirm the family’s eligibility for the study. To give the military parent time to discuss participation in the study with the rest of the family, a follow-up call is scheduled one to two weeks after the screening call. If the family is willing to participate, we ask the military parent to provide informed consent for their own and their children’s participation. We explain to the military parent that, even with the military parent’s formal consent, adolescents must also assent to participate in the research. We contact adolescents independently to obtain their assent only after at least one parent has formally consented to have the adolescents participate in the study.

Once we have affirmed that the family is eligible and the military parent supports their family’s participation, we send a bundle of study packets to the home, one for each person. Each packet contains a printed copy of the appropriate consent or assent documents. Parents’ packets contain three forms: a) consent for their personal participation b) consent for adolescents’ participation; and c) a copy of the assent form that we provide to their adolescents.

Finally, these packets include materials for data collection: paper copies of response cards, instructions for using electronic response cards, instructions to complete the electronic survey and a pre-incentive valued at approximately $5.

#### Adolescent screening

With formal parental consent obtained, we directly reach out to the adolescents, within the time windows and using the method preferred by the parent. Although this initial interaction begins the assent process, it also verifies adolescents’ willingness to have a study team member contact them directly.

On every call with adolescents, before launching into the agenda for the call, we check in with the adolescent about their privacy, encouraging use of earbuds or headphones. If any such concerns surface during the call, the researcher shifts the agenda to finding a time to reschedule the call.

When the privacy assessment is complete, we explain the purpose of the study and the adolescent’s central role in it. We describe the three types of data we are using (survey, interview, and administrative records) and our expectations of families who agree to participate. We offer an opportunity for adolescents to ask any questions.

Finally, we explain how the consent process works. Although we have already talked with their parent, we do not enroll the adolescent unless they want to participate. The adolescent must personally decide whether he or she wants to participate in the study, and the research team must respect that decision. If the adolescent is willing to participate, he or she will need to document that for us. In addition, their parent will need to document her or his permission for the adolescent to participate. Both the adolescent and the parent must agree before the adolescent may participate.

#### Consent and assent

We fully assent adolescents at the beginning of both data collection waves, initially, before they complete their first survey and interview and once again when it is time to complete the second one. When we administer consent or assent, we send the participant a link(s) to the appropriate form(s) and verify that she or he has access. If needed, researchers offer to share a video screen displaying the consent documentation. We review study procedures and talk through the elements of consent as presented in the documents. Within that discussion, we verify their understanding of three types of data we will use in this study (interview, survey, and administrative records). We give all participants another opportunity to ask questions about the study. We also ask them questions to verify their comprehension of the elements of consent or assent. Finally, we ask them whether they are willing to consent to participate in this study, as described below. For both parents and youth, consent/assent is documented electronically in a procedure approved by the IRB. We document participants’ consent/assent by asking them to electronically sign the form HIPAA compliant study portal operated at Purdue.

#### Retention strategies

Evidence-based strategies [52] will be used to minimize attrition between interviews, with particular attention to strategies that have been shown to be effective with youth [53] including: careful attention to educating and motivating participants [54] prompt provision of incentives [55] and effective use of locater data.

#### Compensation

Prospective participants are given a small pre-incentive valued at $1.29. Those screened eligible for participation also are given a preincentive valued at $5 along with their study participation materials. For completing the first survey, each family member receives $15. For completing the first interview, each family member receives $20. Furthermore, when all family members have completed their baseline assessments they are sent a gift valued at no more than $12, which will be something family members can all share (e.g., gourmet popcorn). At the time of the second round of interviews, participants also are sent a gift valued at approximately $5 (e.g., earbuds).

During the second wave of data collection, each family member will be compensated $20 for survey completion and $30 for interview completion, all subject to military regulations for active service members. Compensation will be sent via a separate check for each participant following the completion of each wave. When all family members have completed their second and final wave of data collection, each family member will be sent a project-specific "challenge coin." Each coin costs approximately $7 but has no cash value.

### Data collection procedures

After formal consent/assent is complete, data collection begins immediately. Participants are promptly be sent a link to complete the web-based survey. To facilitate privacy when completing sensitive questions (e.g., perceptions of their relationships with their partner or parents), these surveys are compatible with computers or mobile devices (i.e., tablets and smartphones).

Survey completion requires an internet connection. When this poses a problem, the survey is administered in interview format over the telephone. Interviewers conduct interviews using scripts and prompts built into the interview form and enter participants’ responses directly into Qualtrics’ FedRAMP-compliant data system.

We conduct interviews separately with each participant in a private setting. To establish trust and rapport with the interviewer, we encourage a video connection, but we do not require the participant to have (or use) a camera. If needed, we are prepared to conduct interviews over the telephone. Interviews last approximately 60 minutes for parents and 45 minutes for adolescents and comprise mostly closed-ended questions. We provide participants with response cards: colored cards containing each unique response set that are used in the interview (e.g. 1 = ‘no’, 2 = ‘yes’; 1 = ‘strongly disagree’ to 5 = ‘strongly agree’). Response cards allow participants to answer with symbols instead of words to protect privacy and assist with transitions between sections of the interview when the interviewer references the color needed for the next section.

We provide response cards in electronic format, compatible with tablets and smartphones. To help protect privacy, colors for adolescents are associated with different responses than for adults. Furthermore, when using electronic response cards, adolescents can answer sensitive questions directly on the response cards. When interviewing adolescents, the interviewer and adolescent agree on a word or phrase to use if they want to skip a question, withdraw from the study, or if someone has entered the location where they are completing the interview and provoked a privacy concern.

### Administrative data and measures

Administrative data will be requested from the: **A)** Defense Enrollment Eligibility Reporting System (DEERS)–demographic and background information; **B)** Contingency Tracking System (CTS) and Personnel Tempo (PERSTEMPO)–deployment history data including locations, dates, and types of deployments; and **C)** Medical Data Repository (MDR)–health care utilization and diagnoses for each child and parent.

Measures (see Table 2) to be used for the collection of new data were selected to reflect mechanisms of both resilience and risk, and positive and negative youth outcomes in multiple domains. To support use of latent variables, multiple measures of each major construct were selected for age-appropriateness, established psychometric properties, brevity, and use with military populations and multiple ethnic groups. Measures with established norms or civilian community benchmarks were prioritized (e.g., National Survey of Drug Use and Health, Youth Risk Behavior Survey, National Survey of Children’s Health). Assigned sex of both parents and youth will be included in demographic questions but will be treated analytically as moderators per Aim 3 (sex is operationalized as male and female because of Department of Defense restrictions in place at the time of study approval). Race and ethnicity will be operationalized in accordance with U.S. Office of Management and Budget Guidelines (Office of Management and Budget (OMB) Standards | Office of Research on Women’s Health (nih.gov)). Guided by a family risk and resilience framework, the outcomes of interest will be each child’s social-emotional, behavioral, and academic adjustment. The sequence of measures will be specified, but the order in which parents and children are asked about one another will be randomized to avoid order and fatigue effects.

**Table 2:** Measures to be administered and administrative data to be obtained.

	Measures by Respondent by Instrument	*Data Source*	
*Category / Construct*	*Measure / reliability / reference*	Parent	Youth
**Background Characteristics**			
**1. Demographics**	All: Age, race/ethnicity; Parents: Income, work status, education completed, marital history	Survey	Survey
**2. Family structure**	Household and family structure; age, sex and living arrangements of children; (items asked depends on number of children).	Interview	
**3. Military status & history**	Dates, branch and component, pay grade;	Records	
**4. Civilian Employment**		Survey	
**5. Children’s health**	National Survey of Children’s Health [56]	Interview	
	Pubertal Development Scale(α = .78) [57]		Survey
**6. Personal characteristics**	Emotion regulation [58]	Survey	Survey
	Self-efficacy [59]	Interview	Survey
**Deployments**			
**1. Timing, frequency, duration, and nature.**	Calculated from deployment records using data from DoD Contingency Tracking System and Personnel Tempo files.	Records	
**2. Combat experiences.**	Combat Exposure Scale (α = .85) [60]	Interview	
**Mediating Processes**			
**1. Parents’ Mental Health.**			
***a. Psychological health*:**	Anxiety (GAD-7) (α = .92); [61]	Survey	
	Depression (PHQ-8) (α = .85); [62, 63]	Survey	
	Post-traumatic stress PCL-5 (α = .94); [64, 65]	Interview	
	Spirituality DURAL (α = .78 to .91) [66]	Interview	Interview
	General Life Satisfaction [59]	Survey	
	Meaning and purpose (α>.90) [59]	Survey	
***b. Alcohol use*:**	AUDIT (α = .80); [67, 68]	Survey	
***c. Post-traumatic growth*:**	Post-traumatic Growth Inventory Short Form (10; α = .89);[69]	Survey	
***d. Medical records*:**	MDR records psychiatric diagnoses since deployment: • Mood Disorders (296.0–296.9, • Anxiety Disorders (300.0–300.9), and • Psychoactive Substance Use; 303.0–305.8.	Records	
**2. Parents’ Relationship.**			
***a. Quality of Marriage*:**	Quality of Marriage Index α = .88); [70, 71]	Survey	
***b. Problem Solving*:**	Perceptions of Collaboration (α = .80); [72]	Survey	
	Ineffective Arguing Inventory (α = .86 to .89); [73]	Survey	
***c. Conflict*:**	Conflict Tactics-2 Psychological Aggression (α = .79) [74]	Survey	
**3. Parenting Efficacy.**			
***a. Quality*:**	Parental Acceptance, Rejection, & Control (α = .89-.95); [75, 76]	Survey	Interview
***b. Coparenting*:**	Coparenting Questionnaire (α = .69 to.87) [77]	Survey	
***c. Differential treatment*:**	Perceptions of parental differential treatment (α = .89-.93) [78]		Survey
***d. Monitoring***	Perceptions of monitoring (α = .70 to .77) [79]		Survey
**4. Family Functioning.**			
***a. Overall*:**	Family Assessment Device (General Functioning) (α = .92); [80]	Survey	Survey
***b. Parent child relationship*:**	Items from the National Survey of Children’s Health [81]	Survey	
***c. Sibling relationship*:**	Network of Relationships Inventory (24; α>.70) [82]		Survey
**Moderating Relationships**			
**1. Parents’ Vulnerability Prior to Deployment.**			
***a. Life History***	Family members arriving or leaving; moving; changing schools, Parents: Adverse Childhood Experiences (ACES)	Interview	Interview
***b. Adverse Childhood Experiences***	Parents: Adverse Childhood Experiences (ACES)	Interview	
***c. Family of Origin Functioning***	Subscale of Deployment Risk & Resilience Inventory (α = .85); [83]	Interview	
***d. Psychological symptoms or substance use*:**	*MDR* records for psychiatric diagnoses including Mood Disorders (296.0–296.9, Anxiety Disorders (300.0–300.9), Psychoactive substance; 303.0–305.8;	Records	
**2. Children’s Vulnerability.**			
***a. Adverse experiences*:**	Adverse Childhood Experiences (Child); [81, 84]	Interview	
**3. Social Support.**			
***a. Informal support*:**	Emotional support (α = .91 - .97); [59, 85]	Interview	Interview
	Friendship (α = .91 - .97); [59, 85]	Interview	
***b. Formal support utilization*:**	Access and use of DoD/VA or community services [86]	Interview	
**4. Parental Wounds or Injuries related to Deplyment.**	*MDR records* for *physical injury* E990-E999, Injuries resulting from operations of war, and screening and diagnosis of Traumatic Brain Injury (V15.52l; V80.01).	Records	
**Adolescents’ Outcomes**			
**1. Social-emotional development.**			
***a. Competence*:**	Harter Self-Perception Profile for Children (36; α = .73 - .86); [87]		Interview
***b. Anxiety & Depression*:**	SCARED (α = .74 - .93); [88]		Survey
	Child Depression Inventory– 2 (α = .82); [89]		Survey
***c. Peer relationships*:**	PROMIS peer relationships (α = .92); [90]		Survey
***d. Attachment***	Adolescent Attachment Questionnaire (α = .62 to .80) [91]		Survey
***e. Post Traumatic Growth*:**	PTG Inventory for Children–R (α = .77 - .81); [92]		Interview
***f. Future Expectations*:**	Future Expectations (α = .70) [93]		Interview
***g. Positive Development***	Positive Youth Development (α = .80-.92) [94, 95]		Interview
**2. Behavior.**			
***a. Positive behavior*:**	Flourishing (α = .61);	Survey	
***b. Prosocial & problem behaviors*:**	Strengths and Difficulties Questionnaire (α = .76); [96]	Survey	Survey
***c. Risky behavior***:	Youth Risk Behavior Survey (mean kappa = 60.7%); [97]		Interview
***d. Substance use*:**	National Survey of Drug Use and Health [98] Youth Risk Behavioral Survey 2019 [97]		Survey
***e. Coping*:**	Seeking social support for emotional reasons subscale of the COPE scale (α = .85) [99]		Interview
**3. Academic performance**.	Academic engagement		Interview
	Problems at school [81]	Interview	
**4. Child Health Care Utilization.**	***MDR records*** for lifetime of visits (outpatient and inpatient) with documentation of diagnostic codes (312.0–316.0) with attention to disorders first diagnosed during childhood such as Attention Deficit Hyperactivity Disorder, Learning Disability/Developmental Delay, & Autism and codes (290–319) for commonly diagnosed psychiatric disorders in childhood including (Mood (296.0–296.9 and Anxiety Disorders (300.0–300.9).		Records

### Data management

DMDC used information from DEERS to construct the sampling frame and transmit it to project staff at NHRC. The PII needed by NHRC to create this dataset include electronic data interchange personal numbers (EDIPNs), names, contact information and demographic data, including birthdate. Project staff at NHRC assign TWO key codes to every military parent in the sample: a "field" key code and a "data" key code. Names and contact information needed for data collection, with the field key code as an identifier, are transmitted to the data collection team for recruitment and data collection purposes. EDIPNs and all other identifiers including dates, along with the crosswalk to the data key codes, are retained and kept confidential at NHRC. Contact information along with field key codes are loaded into the data collection operations database to enable the process of recruitment, consent and data collection.

The forms needed for both types of data collection (online surveys and phone interviews) are hosted on the Qualtrics platform. Although the interview team uses these forms, they do not have back-end access to participant survey or interview responses. The only identifier in the Qualtrics data system is the field key code. In addition, none of the PII necessary for recruitment and no PHI will be transmitted to or collected via the Qualtrics system.

Identifying information and participant data never appear or be stored together. To make it possible to match new and archival data, after all data collection for each wave is complete, data sets will be sent to NHRC, merged with DoD archival records, deidentified using National Institutes of Health Guidelines, and–following destruction of the Purdue key code—distributed to investigators for analyses. Once identifiers are removed, other researchers may use the newly collected data. Due to DoD regulations, however, the archival data may not be shared.

#### Data cleaning and preliminary analyses

Prior to analyses, data will be screened for logical inconsistencies and structural problems related to filters and skip patterns, univariate and multivariate outliers, and nonnormality. Each detected problem will be corrected if possible via recoding, data transformation, set to a specific missing code, or left unchanged but flagged. Patterns of missing data will be identified and replaced using archival records where possible. We will check for **introduced bias** associated with interviewer or team, date of interview, question order (e.g., whether mothers or fathers were asked about first), or other factors. When bias is found, that variable will be controlled in subsequent analyses. New timing variables will be constructed, indexed by child age and deployment dates.

#### Psychometrics

Confirmatory factor analysis (CFA) will be used to confirm structures of multi-item measures and measurement equivalence of instruments over time [100]. Cronbach’s alpha [101] also will be calculated. Latent variables will be used when advantageous.

#### Benchmarking

Comparisons will be conducted with established norms or nationally representative community samples, matching participants based on age, sex, education, and minority status wherever possible [102]. For example, exposures to adverse experiences will be benchmarked against responses to the National Survey of Children’s Health, (children) [59] and the CDC Adverse Childhood Experiences data (adults) [103, 104], using items included for this purpose.

### Analyses of aims

Study aims will be evaluated using structural equation modeling with latent variables (SEM) [105, 106]. The SEM framework is flexible, allowing for: 1) simultaneous estimation of multiple equations (i.e., multiple mediators and final outcomes); 2) mixed-effects modeling approaches to nesting of repeated measures within persons and persons within families; 3) use of latent variables to measure constructs independent of random measurement error; 4) estimation appropriate for the complex sample features, including sampling weights and stratified variance estimation [107]; 5) generalized linear modeling capabilities for outcomes with different distributions such as Poisson, binomial, and categorical outcomes [108–111]; and 6) model fit assessment using statistics and indices.

### Model building and estimation

Measurement models will be developed first, per the psychometric analyses. Latent variables will be used when computationally possible. Structural models will be built in stages paralleling study aims, assessing model fit to the data at each stage [112]. Analyses will be conducted using AMOS, Mplus 7 [111], STATA [113], and SAS [114]. Item-missing data will be handled using Full Information Maximum Likelihood (FIML) procedures available within Mplus and STATA [115]^.^ This approach is advantageous, as simulation studies have found it performs superior to listwise deletion and multiple imputation [116]. Sampling weighted estimation that corrects for unequal selection and non-response across strata will be used [107, 117]. In general, an alpha of 0.05 will be used to determine support or lack thereof for hypotheses. However, focus will be on effect size and confidence intervals for the distribution of effects [118].

A mixed-effects modeling approach to the multilevel, nested data structure will be used to explicitly estimate within- and between-family variability [119]. Change in children’s outcomes will be evaluated with two types of models: 1) Three-level mixed-effect models with time nested within child and siblings nested within families, where change is assessed using a predictor variable indicating baseline and follow-up time points, and interactions with the time variable test the effects of predictors on changes in outcomes; and 2) Two-level models with siblings nested in families that include autoregressive effects for each outcome (i.e., control for Time 1 levels), thus controlling for stable levels of each construct and testing effects on residualized change. Both approaches to modeling change provide rigorous inferences about the hypothesized relationships [120]. Models will control for: 1) parents’ age, education, and minority and employment status; 2) family type and size; 3) military branch and paygrade. Sensitivity analyses will be conducted to ensure that these controls do not obscure important findings. Strategies for analyzing each aim are described below.

#### Analyses for aim 1: To assess direct relationships between the timing, frequency, duration and content of siblings’ early exposures to parental deployments and later youth adjustment

Maladjustment is hypothesized to be greater for children whose exposures began earlier (operationalized as child age at first deployment, including gestational age, coded from archival data), were more frequent (i.e., total number of deployments during the child’s lifetime) prolonged (i.e., total days deployed), ended more recently (i.e., days since end of most recent deployment), or who were exposed to deployments where parents’ experiences were more traumatic (i.e., total scores on self-report Combat Exposure items). These hypotheses will be tested using a SEM framework, where a series of path models will estimate the direct effects of deployment characteristics on each indicator of adjustment. Paths from the independent variables will be tested concurrently, thus establishing the unique association for each deployment risk factor. Interactions between different deployment components—frequency, duration, timing, and content—will also be examined to determine whether long-term effects of deployment are exacerbated or mitigated for various deployment combinations. Within-family comparisons will be conducted to determine whether these direct effects differ across siblings.

#### Analyses for aim 2: To assess the role of parents’ psychological health and family processes in mediating relationships between siblings’ early exposures to parental deployment and later youth adjustment

This aim highlights four indirect pathways through which deployment(s) influence youth adjustment. Deployment factors studied in Aim 1 are expected to be negatively related to parents’ psychological health, parental efficacy, marital relationship quality, and family functioning at Time 1, which in turn will be positively related with youth adjustment at Time 2. The magnitude and statistical significance of the indirect effects of deployment through each of these processes will be tested using the INDIRECT command within Mplus, which employs a product of coefficients method of testing mediation [121] and the bootstrap method to compute standard errors for the significance test [122]. Importantly, the four indirect pathways will be tested concurrently, highlighting not only which pathways are critical for understanding the potential enduring effects of parental deployments on youth adjustment, but also the relationships among them. Differences across siblings also will be assessed.

#### Analyses for aim 3: To assess the moderating role of parent and sibling sex, and vulnerability and support in relationship between siblings’ exposures to parental deployment and later youth adjustment

This aim identifies risk and protective factors that may amplify or dampen direct and indirect links between deployments and youth outcomes. Continuous moderators will be tested using procedures outlined by Preacher, Rucker, and Hayes [123]. Specifically, conditional indirect effects will be estimated, with the expectation that relationships between deployment and mediating variables will be stronger when parents experience greater vulnerability and less support, and that relationships between mediating and outcome variables will be stronger when youth experience greater vulnerability and less support. Multi-group analyses will be used to test categorical moderators (i.e., parent and child sex), using likelihood ratio (chi-square) tests to assess whether unconstrained models that allow groups to vary fit better than models constraining direct effects to be equal across groups [105].

### Dissemination

The dissemination plan focuses on participant, scientific, and professional audiences. Reports to participants will include material designed to be of particular interest to youth as well as parents. Submissions will be made to scientific venues including conferences and journals, with both military and nonmilitary foci. For professional audiences, research briefs will be disseminated through the centers and institutes led by our research team, as well as by partner organizations (e.g., National Military Family Association, Military Child Education Coalition, DoD Office of Family Readiness Policy).

## Discussion

### Potential limitations

Although this study is complex and challenging, confidence that it can be successfully populated comes from the large pool of eligible families, the use of recruitment and retention strategies shown to be successful with youth [54], and joint recruitment by military and university partners [124]. Confidence that the study can be successfully implemented comes from the team’s experience with military samples, the data collection techniques to be used, and close coordination with the Naval Health Research Center. Participant agency and confidentiality are being maximized by procedures that use multiple means to assure privacy, allow participants to decline items, and separation of identifying information and data. Participant burden is being minimized by interview strategies that minimize monotony and convey genuine interest in participants’ experiences. Confidence about internal and external validity of study findings is based on probability sampling not limited to families still serving; the inclusion of siblings; data collection and measurement procedures that maximize data quality; careful assessment and management of nonresponse bias and missing data [125]; creative use of archival and new data, and sophisticated analyses that take into account the stratified, multi-level, and longitudinal nature of the data.

### Innovation

The proposed research is unique in its purpose, and its approach improves in multiple ways on existing studies of military children. It is the first to systematically examine the long-term impact of parental deployment with explicit attention to the timing of children’s exposure. It is the first nationally representative study to incorporate multiple offspring per family (i.e., siblings), allowing examination of child-specific effects while controlling for deployment and parent factors [126, 127]. It is designed to include a sufficient number of female service members to permit comparison of mothers’ and fathers’ deployments, and it takes steps to avoid bias toward ‘healthy warriors’ (a bias that can occur in studies of military members because personnel who have been injured tend to leave military service, leaving behind an increasingly distilled population of ‘healthy warriors’), such as including families who have completed their service and stratifying based on early exposure to adversity. It uses archival data and parents’ reports to identify adverse experiences and vulnerabilities in place prior to deployment [128]. Archival records also will provide precise information about frequency, duration, timing, and location of deployments, as well as objective information about medical visits and diagnoses for all participants. Unlike most research on military families, it explicitly attends to both positive and negative outcomes for children, not just in relation to parental deployments, but also in relation to caravans of support or adversity that may accompany them. Multiple equation modeling will allow estimation of variability both within and between families over time, account for dependencies of within-family data, and measurement error and missing values inherent in surveys, and correct for unequal probabilities of sample selection [86, 129, 130].

This study will identify downstream consequences of early exposure to parental deployment during a key developmental period and contribute to literature about children’s risk and resilience in the family context. It will generate insights about how military-connected children compare to national samples of children on key outcome variables, taking exposures to adversity into account. It will contribute to understanding the extent to which military-connected children’s outcomes during adolescence are accounted for by characteristics of parental deployments they experienced early in life vs. their own and their parents’ early exposures to other adverse experiences. It will reveal within-family variations in military children’s outcomes and their antecedants. It will identify which risk and resilience processes appear to be most important for particular adolescent outcomes among children exposed early in life to parental military deployments, and how these effects differ as a function of parent and child assigned sex.

The implications will be important for prevention programs in DoD, schools, health care providers, and community organizations as they consider how best to optimize long-term outcomes for children.

### Status and timeline

Regulatory approval to collect data has been received and data collection has begun. Data collection and sample acquisition are scheduled to continue for 39 months, followed by analyses, reports and dissemination for 13 months.
